# Video Narratives Intervention Among Stroke Survivors: Feasibility and Acceptability Study of a Randomized Controlled Trial

**DOI:** 10.2196/17182

**Published:** 2020-07-10

**Authors:** Jamuna Rani Appalasamy, Joyce Pauline Joseph, Siva Seeta Ramaiah, Anuar Zaini Md Zain, Kia Fatt Quek, Kyi Kyi Tha

**Affiliations:** 1 School of Pharmacy Monash University Malaysia Bandar Sunway, Selangor Malaysia; 2 Jeffrey Cheah School of Medicine and Health Sciences Monash University Malaysia Bandar Sunway, Selangor Malaysia; 3 Department of Neurology Hospital Kuala Lumpur Ministry of Health Kuala Lumpur Malaysia; 4 Medical Department Subang Jaya Medical Center Sunway Malaysia

**Keywords:** feasibility and acceptability, medication understanding, use self-efficacy, stroke, video narratives

## Abstract

**Background:**

A large number of stroke survivors worldwide suffer from moderate to severe disability. In Malaysia, long-term uncontrolled stroke risk factors lead to unforeseen rates of recurrent stroke and a growing incidence of stroke occurrence across ages, predominantly among the elderly population. This situation has motivated research efforts focused on tapping into patient education, especially related to patient self-efficacy of understanding and taking medication appropriately. Video narratives integrated with health belief model constructs have demonstrated potential impacts as an aide to patient education efforts.

**Objective:**

The aim of this study was to investigate the feasibility and acceptability of study procedures based on a randomized controlled trial protocol of a video narratives intervention among poststroke patients. We also aimed to obtain preliminary findings of video narratives related to medication understanding and use self-efficacy (MUSE) and blood pressure control.

**Methods:**

A parallel group randomized controlled trial including a control group (without video viewing) and an intervention group (with video viewing) was conducted by researchers at a neurology outpatient clinic on poststroke patients (N=54). Baseline data included patients’ sociodemographic characteristics, medical information, and all outcome measures. Measurements of MUSE and blood pressure following the trial were taken during a 3-month follow-up period. Feasibility of the trial was assessed based on recruitment and study completion rates along with patients’ feedback on the burden of the study procedures and outcome measures. Acceptability of the trial was analyzed qualitatively. Statistical analysis was applied to ascertain the preliminary results of video narratives.

**Results:**

The recruitment rate was 60 out of 117 patients (51.3%). Nevertheless, the dropout rate of 10% was within the acceptable range. Patients were aged between 21 and 74 years. Nearly 50 of the patients (>85%) had adequate health literacy and exposure to stroke education. Most of the patients (>80%) were diagnosed with ischemic stroke, whereby the majority had primary hypertension. The technicalities of randomization and patient approach were carried out with minimal challenge and adequate patient satisfaction. The video contents received good responses with respect to comprehension and simplicity. Moreover, an in-depth phone interview with 8 patients indicated that the video narratives were considered to be useful and inspiring. These findings paralleled the preliminary findings of significant improvement within groups in MUSE (*P*=.001) and systolic blood pressure control (*P*=.04).

**Conclusions:**

The queries and feedback from each phase in this study have been acknowledged and will be taken forward in the full trial.

**Trial Registration:**

Australian New Zealand Clinical Trials Registry ACTRN 12618000174280; https://www.anzctr.org.au/Trial/Registration/TrialReview.aspx?id=373554

## Introduction

### Background

Establishing a patient narrative is a common method for analyzing how individuals with illnesses express themselves to best recognize and reflect the values and teachings that are most important to them and how they react toward their actions [1]. Personal and interpersonal factors such as coping strength and family or social support form the basis of these narratives [2]. Thus, a narrative can influence changes in health behaviors toward achieving appropriate health outcomes [3,4]. Narratives incorporated in multimedia format can effectively deliver patients’ stories to viewers who can then become “carried away” by their peers’ experiences and help them to learn from others [5]. A high percentage of the delivered information in patient education offers engagement via the visual and hearing senses. Therefore, the use of video narratives offers a great chance of proper comprehension and reflection among patients [6].

Video narratives have long been explored and developed for various patient education purposes in chronic disease management [7-9]. However, there are limited studies on video narrative-based interventions in poststroke patients, and their outcome measures varied in terms of the severity of the disease and psychosocial challenges [10,11]. In Malaysia, the ischemic stroke incidence has shown an increase of approximately 30% annually, with an increase of approximately 19% for hemorrhagic stroke, which is a more prominent condition among the aging community [12,13]. The majority of poststroke patients experience physical disability, learning, and speech impairment, which also lead to emotional problems [14,15]. Hence, an individual who experienced stroke will benefit from resilience, which requires self-efficacy [16,17]. Medication nonadherence had been associated with a lack of self-efficacy in poststroke patients, especially with regard to understanding and taking medication [18]. Thus, patient education efforts that focus on enhancing medication understanding and use self-efficacy (MUSE) are warranted. Indeed, sustaining medication adherence is crucial to achieve optimal recurrent stroke treatment effects [19]. Despite the advancement of stroke prevention treatment, medication nonadherence prevalence remains notable among patients with high stroke risk factors such as hypertension and cardiac disease [20,21]. Social learning theory explains that a person’s behavior depends on adaptation of their thoughts and beliefs, which are influenced by the environment and in turn control the individual’s actions [22]. This theory proposes that medication adherence relates to an individual’s perception of health issues, which influences self-efficacy toward prescribed medication [23,24].

There are limited studies aimed at understanding the use of video narratives with the integration of health belief constructs and motivational cues. In addition, little is known about the effect of video narratives on poststroke patients in particular. Stroke survivors require motivational support, which could help them to enhance their effort in understanding prescribed medication and taking it appropriately [25]. We believe that video narratives offer an opportunity to facilitate the existing stroke patient education effort of the medication therapeutic adherence clinic (MTAC) [26]. Moreover, the outpatient clinic waiting time and area offer a potential period and venue for patients to receive these inputs [27]. Thus, we aimed to evaluate the feasibility and acceptability of a video narratives randomized controlled trial (RCT) among poststroke patients in Malaysia.

### Objectives

This study was an a priori phase of a powered RCT [28] focused on determining the recruitment, retention, and completion rate of the trial. Patients’ qualitative feedback and views were collected with respect to the acceptability of the videos. We also analyzed the preliminary changes of MUSE over the course of the intervention and compared the findings with a control group.

## Methods

### Ethical Considerations

The study received ethics approval from the Malaysian Medical Research and Ethics Committee, Ministry of Health Malaysia (NMRR ID-15-851-24737) and the Monash University Human Research Ethics Committee (ID 9640).

### Sample Size, Eligibility, and Randomization

Given the lack of similar studies, there was no referral for appropriate effect sizes. Moreover, a feasibility study without inferential results does not necessarily require a power analysis [29]. Therefore, we estimated the sample size based on practical considerations and experience of the researchers [30,31]. This pretest and posttest design, two-arm RCT was conducted from March 2018 to June 2018 among informed and consenting stroke survivors who had clinic appointments at the Neurology Outpatient Department of Hospital Kuala Lumpur (HKL), Malaysia. We aimed to recruit a minimum of 25 patients per group. Eligible and consenting patients were adults diagnosed with their first stroke within 6 months of the recruitment period, and were prescribed stroke risk preventative medications from HKL. Those excluded were diagnosed with depression (Patient Health Questionnaire score≥1) and cognitive impairment (Montreal Cognitive Assessment score<26). We only included patients who could comprehend the English or Malay language.

Randomization was performed via the block method between 2, 4, and 6 lengths placed in opaque envelopes. The allocations of each block were also randomized. Patients were either allocated to the standard care (control) or intervention (with video viewing) group. The full description of the study’s methodology is available in our protocol trial report [28].

### Video Narratives

Based on research interest and considering the motivational need of poststroke patients, we developed a set of video narratives incorporated with health belief model constructs. The validation procedures and narrative contents in the English and Malay languages have been described in detail in our previous paper [32]. The video narratives provided messages (culturally appropriate for the local context), which served as triggers to motivate patients to be resilient in attaining self-efficacy skills as per their perceived needs. To reflect the purpose of role models, a neurologist and a stroke survivor volunteered to narrate their story in a video to render their honest emotion while stressing the need to adhere to stroke preventative medication (see Multimedia Appendix 1 and Multimedia Appendix 2). Short quotes and subtitles were incorporated to increase attentiveness toward the comprehension of their messages [33].

### Intervention Design and Study Procedures

**Figure 1:**
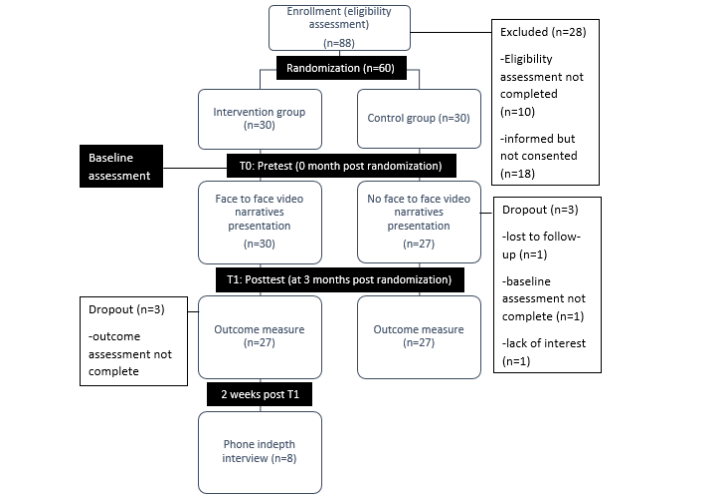
CONsolidated Standards Of Reporting Trials (CONSORT) flowchart of poststroke patients throughout the study.

The groups in this RCT received treatment with ongoing patient education and counseling as per HKL neurologists’ recommendations. The treatment compliance practice included MTAC appointments, self-monitoring checks, and outpatient clinic attendance. Both groups received pamphlets on stroke awareness and its preventative medication information, and the “teach-back method” was used to help reduce discrepancies between the two groups [34,35]. The “teach-back” queries were related to medication dose, frequency, indication, and time as recommended by the MTAC. In addition to this standard care, only the intervention group received face-to-face video narratives. Figure 1 illustrates the CONsolidated Standards Of Reporting Trials (CONSORT) flowchart showing patients’ participation throughout the study at data collection time points. We collected the quantitative data at baseline (T0) and 3 months postrandomization (T1), and collected qualitative data via a semistructured interview upon completion of the study. Similar data collection and follow-up procedures as applied in the main study protocol were followed [28]. Blinding was impossible for the patients. This also includes the researchers who conducted the assessment of the questionnaire, except for the treating neurologists.

### Outcome Measures

#### Main Outcome Measure

The main outcome measure assessed at pretest (T0) and posttest (T1) referred to MUSE. The MUSE is assessed on an 8-item Likert-type scale with total scores ranging from 0 to 32, which measures the perceived self-efficacy in understanding and taking prescribed medication. It has good internal consistency (Cronbach α >.70) with adequate construct and predictive validity [36]. We repeated MUSE for each prescribed stroke preventative medication.

Understanding and taking medication is also associated with other factors such as knowledge, perception, or belief [37]. Therefore, we aimed to observe changes in these factors as well. Hence, the feasibility study assessed the baseline of the following secondary outcome measures.

#### Stroke Knowledge Test

The stroke knowledge test of 20 items is a measure of stroke knowledge, which is vital in evaluating the quality of stroke education modules. The stroke knowledge test has received acceptable and favorable ratings from health and educational experts, which reflected its excellent reliability and construct validity [38].

#### Brief Illness Perception Questionnaire

The brief illness perception questionnaire (BIPQ) is assessed on a 9-item Likert scale with a 0 to 10 scoring range that intends to evaluate the perceptive and emotive illustrations of the effect of an illness on a patient. This measure demonstrated good test-retest reliability and high concurrent and discriminant validity [39].

#### Belief About Medicine Questionnaire

Belief About Medicine Questionnaire (BMQ) is an 18-item questionnaire with two constructs: BMQ-General and BMQ-Specific. Both constructs are further divided into the subscales of overuse harm and necessity concerns. The scales of both constructs have acceptable internal consistency, discriminant validity, and reliability [40]. This measure assesses general and specific medication beliefs and perceptions. Nevertheless, only the BMQ-Specific construct measure was repeated for each prescribed stroke preventative medication in this study.

#### Short Form (36) Health Survey

Short Form (36) Health Survey (SF-36) comprises a 36-item questionnaire with scoring between 0 and 100. The SF-36 is able to measure perceived physical and mental constraints. It has been translated, validated with acceptable psychometric properties, and used widely in various clinical settings to measure the overall health state [41].

#### Other Measures

Other measures included systolic and diastolic blood pressure, fasting blood glucose, total cholesterol, and international normalized ratio.

We received the “permission to use” from the authors of the original (English) and the translated (Malay language) versions of the questionnaires, deemed to be locally appropriate for this study [42-45]. The researchers also conducted a face and content validity analysis with 5 patients and experts prior to this study to confirm the understandability and the content validity index>0.80 of MUSE, BMQ-specific, and BIPQ for the named medication(s), for which the word “illness” was replaced with “stroke” in the measures.

### Data Collection Procedures

We obtained the patients’ sociodemographic and health information using a data collection form that recorded gender, age, ethnicity, educational attainment, and health literacy status using the Newest Vital Sign format [46]. Concurrently, we retrieved clinical health data on the type of stroke and stroke risk factors from the hospital’s patient medical records. All consenting patients from the intervention group had the option to volunteer to participate in a 10-minute phone or face-to-face interview within 2 weeks of follow up from the data collection point (T1). This was conducted to obtain feedback on the burden of outcome measures (questionnaires) and the acceptability of viewing the video as an intervention. The researchers maintained data confidentiality and patients’ safety as per the protocol [28].

### Data Analysis

#### Quantitative Data

Statistical analyses were performed using IBM SPSS Statistics V.24.0 with *P*<.05 as the threshold significance level. Descriptive statistics (eg, means and percentages) were used to describe the characteristics of both the control and intervention groups, along with the study and intervention’s feasibility and acceptability. Chi square tests were applied to explore dissimilarities in patient characteristics between both groups at baseline. Data differences over time between the two time points (T0 and T1), at intergroup and intragroup levels, were also analyzed for the outcome measures using the Mann-Whitney *U* test and the Wilcoxon rank-sum test. The results for outcome measures and mean differences were calculated as means (SD), range, or 95% CI as appropriate. Multiple imputation was applied for missing data.

#### Qualitative Data

The phone interview recordings were transcribed and translated verbatim. Two researchers reviewed the transcripts, wherein they occasionally met to discuss the developed themes. The themes were then verified by another researcher to assure uniformity and quality. The transcripts and written feedback were analyzed using thematic analysis [47]. We applied the software NVivo 11 (qualitative data analysis software; QSR International Pty Ltd, Version 11, 2015) to identify the themes and to help in organizing the codes.

## Results

### Participant Characteristics

Table 1 presents the sociodemographic and health information of patients who participated in and completed the trial over the full study period. Both groups comprised more men than women with a dominance of more than 50%, and most patients were predominantly of Malay ethnicity in both groups (>80%). Patients were between 21 and 74 years old, with a mean age of 56 years (SD 13.1) for the control group and 53 years (SD 11.6) for the intervention group. Nearly 50 participants (>85%) had secondary education, which included tertiary attainment, with adequate health literacy and exposure to stroke education. More than half of the patients were unemployed. The majority of patients (>80%) had experienced ischemic stroke and had several underlying stroke risk factors inclusive of hypertension, but not all controllable risk factors were documented, such as diet, obesity, and physical inactivity, due to lack of data in medical records. Among them, approximately 25 patients (50%) had diabetes, and more than 50 patients (about 90%) were taking at least three types of stroke preventative medication. There were no significant differences in sociodemographic characteristics and measures between the two groups, except for gender.

The following sections are presented as per study objectives and with subdivisions to the feasibility and acceptability of (1) the RCT procedures, (2) video narratives intervention, and (3) preliminary findings of the effect of the video narratives on MUSE and blood pressure as a stroke risk factor control.

**Table 1:** Sociodemographic and health data of patients at 3-month follow up (N=54).

Characteristic		Control (n=27)	Intervention (n=27)
**Gender, n (%)**			
	Male	18 (67)	15 (56)
	Female	9 (33)	12 (44)
**Age (years), n (%)**			
	≥60	10 (37)	8 (30)
	40-59	13 (48)	16 (59)
	≤39	4 (15)	3 (11)
Age (years), mean (SD)		56 (13.1)	53 (11.6)
**Ethnicity, n (%)**			
	Malay	22 (82)	23 (85)
	Chinese	1 (4)	1 (4)
	Indian	4 (15)	3 (11)
**Education attainment, n (%)**			
	Primary	4 (15)	2 (7)
	Secondary	15 (56)	18 (67)
	Tertiary	8 (30)	7 (26)
**Health literacy level, n (%)**			
	Adequate	23 (85)	24 (89)
	Limited	4 (15)	3 (11)
**Employment status, n (%)**			
	Employed	11 (41)	7 (26)
	Unemployed	16 (59)	20 (74)
**Type of stroke^a^, n(%)**			
	Ischemic	22 (82)	25 (93)
	Hemorrhagic	0 (0)	0 (0)
	TIA^b^	5 (18)	2 (7)
**Stroke risk factors (comorbidities), n (%)**			
	Hypertension and other risks^c^	24 (89)	26 (96)
	Diabetes only	2 (7)	1 (4)
	Other risks only	1 (4)	0 (0)
**Varieties of prescribed medication, n (%)**			
	≤2 types	2 (7)	3 (11)
	≥3 types	25 (93)	24 (89)
**Received formal or informal information about stroke prevention, n (%)**			
	Yes	23 (85)	24 (89)
	No	4 (15)	3 (11)

^a^Inclusive of modifiable stroke risk factors other than hypertension (eg, diabetes, heart diseases, hyperlipidemia, current smoking/alcohol).

^b^TIA: transient ischemic attack.

^c^Other risks include nonspecific International Classification of Diseases stroke codes.

### Feasibility and Acceptability of the RCT Procedures

The randomizing method, administration, and questionnaire retrieval at the outpatient waiting zone were effectively carried out. We experienced minimal challenges and uninterrupted flow at ushering patients individually to an allocated quiet room for video viewing. We received written feedback from 12 patients. Overall, the patients were satisfied with the study procedures, including the usage of a 5.3-inch-wide screen tablet and headphones, but commented on the burden of the self-administered questionnaires (for assessing the outcome measures). A few remarks were related to the exhaustive repetition of the MUSE and BMQ for each type of medication and the extensive length of the SF-36. Furthermore, there were suggestions to receive a token of appreciation for sustaining their participation.

#### Recruitment Rate

A total of 117 poststroke patients were screened from clinical records within 1 month for recruitment of trial participation, but only 88 patients were eligible according to the inclusion and exclusion criteria, resulting in an eligibility rate of 75.2%. Among all 88 patients, 70 patients provided consent to participate, but only 60 of them completed the baseline assessment. Hence, the recruitment rate was 51.3%. The most common reasons that patients declined enrollment were a language barrier, afraid of increased stress, and refusal.

#### Dropout and Study Completion Rates

During the baseline assessment (T0) and the 3-month follow-up assessment (T1), the number of patients completing the study dropped to 54 from 60 (90%), which reflected a dropout rate of 10%. The most common reason for not completing the study was an inability to be contacted, which we considered to indicate refusal for further participation.

### Feasibility and Acceptability of the Video Narratives Intervention

We sought to gain in-depth information on technical issues and views on the video narratives’ usefulness as a motivational trigger to improve MUSE. The results of several subthemes identified are presented in Textbox 1.

**Themes and quotes associated with the feasibility and acceptability of the video narratives intervention.**

**Feasibility of the video narratives**

**Main theme: Engagement and comprehension**

- Messages were short, transparent, and easily understood
  You must not make the video too long. Like this one, that is just nice…not boring….What you see on video, the doctor was very good
  P2
- Patients had the option to view it in their preferred language; either in English or in Malay
  Things related to stroke should be explained by patients themselves… not just knowledge but experience… so that others will be aware
  P6
- The narratives were suitable for the elderly
  Most elderly patients are very stubborn about taking medicine. Show the video especially to the elderly patients
  P7
- Appropriate video viewing frequency
  Watching the video once in a while like this is good….
  P3

**Main theme: Generalizability**

- There were suggestions to share the video among friends via other media platforms such as WhatsApp or to continuously play it on air in the hospital.
  I can share this video with my friends in WhatsApp
  P1
  I want to send the video for my friends to watch!
  P5
  Maybe what they can do probably is over some TV set… what do you call that…program? Put the show, I mean like this type of video, what’ll happen when you have a stroke and all that… So that patients can listen instead of giving TV1 all the time you know?
  P2

**Acceptability of video narratives**

**Main theme: Informative and reminder**

- The videos narratives were a “trigger” toward proactivity and enhanced patients’ awareness about stroke and its preventative treatment.
  They remind us of important medicine… They remind us of the danger of the second stroke… to take medicine well and to have a healthy lifestyle
  P7
  Helpful….more understanding about stroke
  P6
  Awareness… before that we were not really concerned about our health. Now, after the advice it’s different... like a guide
  P4
  Patients can recover from stroke and (it) won’t recur if we take the medicine prescribed by doctors according to the right schedule on time
  P2

**Main theme: Emotional consolation**

- Viewing the video narratives provided some hope and less fear to overcome stroke challenges.

It’s a bit of both worrying and confidence… There is always a worry about what can happen, but it also gives you an idea (on) what to do, and what to be careful, and what to be aware

P3

- The video was an aid to their plight that there was life after stroke.
  Because you are a stroke patient, you have to look at the guidelines… you want to know (more)… you have to take care of yourself, right? You’ll be confident when you have such thing (to guide you) … Before this… you don’t know anything… fear about getting another attack… right?
  P1
  Others must know that people who got stroke, just like us, but they can recover. Sometimes, for stroke, people can’t really help, except for the patients themselves
  P4

**Main theme: Perception and confidence**

- The motivational cues inspired the patients and raised confidence among themselves.
  … (sharing) someone’s experience to change others’ mind. Sometimes, we need to listen to their stories for us to make a change
  P4

- They had a positive outlook towards stroke recovery and were willing to do better to improve their health condition.
  I feel that I have to follow the advice, for example, taking medicine, doing blood test… that have been mentioned… (The videos) seem to inspire us to take care of health so that we won’t get sick. Perhaps to give encouragement makes me feel that I can recover from stroke if follow all the advice
  P5
  Usually, if you never had a stroke before, you don’t really care about watching the videos. Once you had (a stroke), you’ll realize that… health is important… you have to take care of it… watch their story… that’s it!
  P1
  Now I ask my doctor more questions if I don’t understand….
  P2

### Preliminary Findings

Table 2 presents the results as per the trial protocol of outcome measures at T1 for MUSE and blood pressure control. There were no significant differences in outcome measures at baseline (T0) between the two groups (*P*<.001). All patients were on antiplatelet therapy, and the majority of patients diagnosed with hypertension were on antihypertensive medication (control group, n=24; intervention group, n=26). Therefore, only the general MUSE and specific MUSE for antithrombotic and antihypertensive medications were applied.

Both groups showed improvement in MUSE scores, but the intervention group presented greater differences from baseline compared to the control group. Similar trends were found for blood pressure control, whereby the intervention group had better systolic pressure regulation compared to the control group (Table 3). The MUSE outcomes of the intervention group were significantly different for the between-group and within-group analysis (Table 4).

**Table 2:** Outcome measurement of both groups at baseline (T0^a^) and posttest (T1^b^) assessments.

Measure			Control group, mean (SD), range				Intervention group, mean (SD), range		
		T0		T1		T0		T1	
**MUSE^c^**									
	All medications	27.0 (4.71), 16-32		27.6 (3.76) 20-32		26.3 (5.81), 16-32		30.1 (3.62), 20-32	
	Hypertensive^d^	36.2 (4.62), 17-32		27.8 (35.7), 22-32		26.3 (5.59), 16-32		30.1 (3.51), 20-32	
	Antithrombotic^e^	27.0 (4.70), 16-32		24.4 (3.73), 20-32		27.3 (5.43), 16-32		30.0 (3.57), 20-32	
Systolic blood pressure^d^ (mmHg)		138.7 (7.84), 127-162		137.9 (10.78), 124-60		147.0 (16.8), 121-186		137.8 (12.74), 117-165	
Diastolic blood pressure^d^ (mmHg)		79.6 (11.62), 54-107		80.0 (10.93), 60-105		85.7 (11.59), 58-109		85.0 (9.07), 68-100	

^a^T0: baseline (control group n=27, intervention group n=30).

^b^T1: 3 months postrandomization (control group n=27, intervention group n=27).

^c^MUSE: medication understanding and use self-efficacy.

^d^Prescribed with antihypertensive medication and diagnosed with hypertension as a primary factor (control group n=24, intervention group n=26).

^e^Prescribed antithrombotic medication as a prerequisite preventative treatment for stroke (control group n=27, intervention group n=27).

**Table 3:** Comparison of outcome measurement within groups.

Measure		Control group			Intervention group			
		T1^a^ – T0^b^ (95%CI)	*Z* value	*P* value^c^		T1 – T0	*Z* value	*P* value^c^
**MUSE^d^**								
	All medications	0.52 (–1.61-2.65)	–0.85	.39		4.57 (1.94-7.19)	–3.63	.001
	Hypertensive^e^	1.26 (–0.36-2.89)	1.79	.07		4.35 (2.18-6.52)	–3.60	<.001
	Antithrombotic^f^	–0.91 (–1.29-3.11)	–0.37	.71		3.70 (1.62-5.77)	–3.18	.001
Systolic blood pressure^e^ (mmHg)		–1.87 (–6.70 to –2.96)	0.54	.59		–13.04 (–22.22 to –3.87)	–2.03	.04
Diastolic blood pressure^e^ (mmHg)		0.48 (–6.17-7.13)	–0.59	.56		–0.87 (–6.58-4.84)	–0.144	.89

^a^T1: baseline (control group n=27, intervention group n=30).

^b^T0: baseline (control group n=27, intervention group n=30).

^c^Wilcoxan signed-rank test

^d^MUSE: medication understanding and use self-efficacy.

^e^Prescribed with antihypertensive medication and diagnosed with hypertension as a primary factor (control group n=24, intervention group n=26).

^f^Prescribed antithrombotic as a prerequisite preventative treatment for stroke (control group n=27, intervention group n=27).

**Table 4:** Comparison of outcome measurement between the control and intervention groups.

Measure		Difference in T1^a^ (95%CI)	*Z* value	*P* value^b^
**MUSE^c^**				
	All medications	2.74 (1.29-4.19)	–3.14	.002
	Hypertensive^d^	2.35 (0.87-3.81)	–2.65	.008
	Antithrombotic^e^	2.78 (1.28-4.29)	–3.14	.002
Systolic blood pressure^d^ (mmHg)		0.96 (–6.58-8.49)	–0.17	.86
Diastolic blood pressure^d^ (mmHg)		6.04 (0.94-11.14)	–1.84	.07

^a^T1: mean score/measurement differences between intervention and control groups at 3 months postrandomization.

^b^Mann-Whitney U test.

^c^MUSE: medication understanding and use self-efficacy.

^d^Prescribed with antihypertensive medication and diagnosed with hypertension as a primary factor (control group n=24, intervention group n=26).

^e^Prescribed antithrombotic as a prerequisite preventative treatment for stroke (control group n=27, intervention group n=27).

## Discussion

### Principal Findings

The aim of this study was to assess the feasibility and acceptability of a planned intervention in an actual clinical setting. We successfully tested the intervention processes as per the trial protocol from the initial stage of recruitment, randomization, baseline assessment, and at the first outcome phase.

#### Feasibility and Acceptability of Study Procedures and Outcome Measures

The recruitment period of 1 month was found to be appropriate as we were able to enroll more than the minimum planned sample size. The recruitment rate of 51.3% was comparable to the average trend of stroke trials conducted from 1990 to 2014, whereby there were no substantial increase or decline rates over the past 25 years [48]. At 3 months, the attrition rate was below the a priori projection of 15%. The positive recruitment rate might reflect concerns and interest to enhance stroke recovery. Nonetheless, we believe that more effort would be needed to sustain the dropout rate expected for the full 12-month trial, as reflected by the desire for monetary compensation indicated by a few patients. Despite this, we found that poststroke patients were able to cope with the study flow, and the extent of participation persuasion was not coercive, which was within the trial ethics jurisdiction and funding capacity [49].

The repeated MUSE on each stroke preventative medication group was necessary to elicit a significant association of self-efficacy with medication categories. However, there were concerns that the questionnaire administering process was time-consuming and created a feeling of redundancy among the patients. As the majority of poststroke patients were primarily hypertensive [50], it was crucial to obtain responses from patients within the three categories in the full trial. Owing to the inability to recruit more samples with other primary diagnosed stroke factors such as diabetes and hyperlipidemia, it remains to be investigated whether an influx of broader inclusion criteria would change the patient sample proportion. Similarly, comments related to the SF-36 received were similar with respect to the burden of its lengthiness. Nonetheless, it was not possible to substitute this questionnaire with other versions [51] due to the constricted contract for the Malay language version. Therefore, with all these issues taken into consideration, the full trial protocol was carried out as planned without significant changes in its outcome measures and study procedures.

#### Feasibility and Acceptability of Video Narratives

The video contents were comprehensible (layman terms) and had a sensible touch of emotion suitable for the local culture and language with a clear benefit for aging poststroke patients. There was a rising awareness of how audio-visual technology can influence different age groups and social environments. This feedback was comparable to similar trials with positive outcomes [52-54]. Nevertheless, face-to-face video viewing was maintained in this trial to prevent restriction of sample inclusion and exclusion criteria.

It was not surprising that the videos were perceived to be motivational for the poststroke patients. The qualitative results showed positive responses, which increased our anticipation that the videos incorporated with health belief constructs could facilitate standardized ongoing patient educational efforts in a clinical setting. The concise keywords used as cues added with authentic emotions triggered awareness and inspiration among patients toward being more self-efficacious in understanding and taking medication appropriately. Recent studies in different settings and samples have reported similar findings [55,56]. Other than that, educational video narratives could also improve the doctor-patient relationship. Paralleling previous studies, the combination of both personal views of the doctor and patient in this study potentially caused small positive perception changes in MUSE or initiated regular health monitoring [57,58]. Therefore, the preliminary outcomes in the intervention effectiveness analysis corresponded with our justification.

#### Preliminary Findings

In an associated review, it was clear that the presentation of “real people” has a motivating effect for peers with similar underlying illness [26] (stroke in this case). Hence, the initial impact on MUSE and systolic blood pressure provides insights toward a purposeful trial. The 3-month gap of video viewing to moderate the burden and the dropout rate was also appropriate, as indicated in a previous study [55]. Thus, we concluded that the measurement of self-efficacy among poststroke patients at the per allocated period could be assessed effectively [59]. Furthermore, these results were consistent with studies indicating a significant improvement in MUSE, which paralleled improved stroke risk factors control, such as systolic blood pressure [60,61]. Nevertheless, as we observed variation (coefficient of variation>1) for all variable differences with inconsistent confidence intervals, a bigger sample size would confirm its significance; otherwise, these positive results would have to be interpreted judiciously.

### Strengths and Limitations

In designing the study, we spared no effort at not disrupting the workflow of a real-life outpatient clinic environment. However, there were unavoidable circumstances. For example, the blood pressure measurement was to be carried out by the physician or neurologists in their clinic only. We also foresee an issue since individual follow-up of neurology outpatient clinic appointment dates varied from 2 to 5 months and coincided with other clinical appointments (eg, diabetes clinic, heart disease clinic, rehabilitation, physiotherapy, and MTAC). Therefore, it was appropriate to consider documenting blood parameters at T0, T2, and T4 for the intervention effectiveness analysis. Hence, it was a challenge to ensure patients to view the videos within the 3-month gap from baseline. Nevertheless, we overcame these issues with transport reimbursement provided to the patients so as to maintain the retention rate and self-posted questionnaires to avoid further loss of data.

Several other limitations are the exclusion of patients who were unable to comprehend the English and Malay languages, which would have increased bias and limited the generalizability of the preliminary results. In addition, as a cost-effective approach, we were not able to carry out this study for more than 3 months. Despite all these limitations and challenges, the study procedures and outcome measures strategy were considered to be robust to inform the design of a successful 1-year RCT [28]. This study demonstrated versatile and helpful methods in achieving unanimous consensus.

### Conclusion

Preliminary studies are crucial in assessing the success of a novel intervention [62]. This innovative method has been applied in various clinical settings in developed countries [52,63]. However, it has not yet been investigated for the poststroke patient population in Malaysia. This study successfully assessed the feasibility and acceptability of the video narrative intervention. The feedback and lessons learned from the baseline until the first follow-up assessment increased the awareness of both foreseen and unforeseen challenges. More importantly, we tested the initial requirement for full RCT accomplishments such as patient recruitment, feasibility, and acceptability of all outcome measures. Future research on the effectiveness of using culturally appropriate video narratives for a more extended period is warranted.

## Appendix

Multimedia Appendix 1 Screenshot of video narratives of neurologist.

Multimedia Appendix 2 Screenshot of video narratives of patient.

Multimedia Appendix 3 CONSORT checklist.

## Abbreviations

BIPQ: Brief illness perception questionnaire
BMQ: Belief about medicine questionnaire
CONSORT: CONsolidated Standards Of Reporting Trials
HKL: Hospital Kuala Lumpur
MTAC: medication therapy adherence clinic
MUSE: medication understanding and use self-efficacy
RCT: randomized controlled trial
SF-36: Short Form (36) Health Survey
T0: baseline
T1: 3 months postrandomization
