# Supplementary figures and images for: Video Narratives Intervention Among Stroke Survivors: Feasibility and Acceptability Study of a Randomized Controlled Trial
Source: JMIR Aging. 2020 Jul 10;3(2):e17182. doi: 10.2196/17182 (PMC7382013; doi:10.2196/17182)

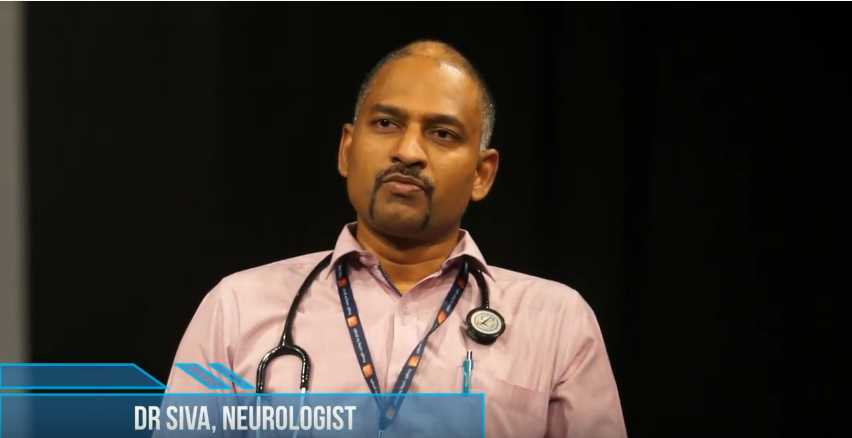

Supplement: Multimedia Appendix 1 [file aging_v3i2e17182_app1.png]

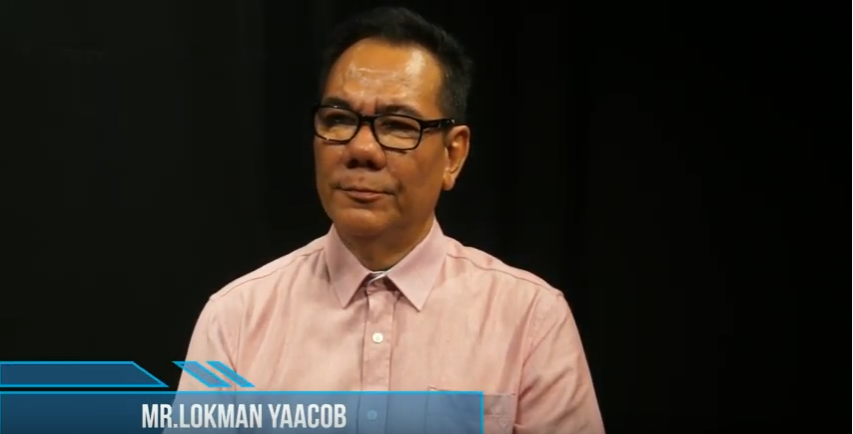

Supplement: Multimedia Appendix 2 [file aging_v3i2e17182_app2.png]
